# Rotating lattice single crystal architecture on the surface of glass

**DOI:** 10.1038/srep36449

**Published:** 2016-11-03

**Authors:** D. Savytskii, H. Jain, N. Tamura, V. Dierolf

**Affiliations:** 1 Materials Science and Engineering Department, Lehigh University, Bethlehem, PA, 18015, USA; 2 Lawrence Berkeley National Laboratory, Berkeley, CA, 94720, USA; 3 Physics Department, Lehigh University, Bethlehem, PA, 18015, USA

## Abstract

Defying the requirements of translational periodicity in 3D, rotation of the lattice orientation within an otherwise single crystal provides a new form of solid. Such rotating lattice single (RLS) crystals are found, but only as spherulitic grains too small for systematic characterization or practical application. Here we report a novel approach to fabricate RLS crystal lines and 2D layers of unlimited dimensions via a recently discovered solid-to-solid conversion process using a laser to heat a glass to its crystallization temperature but keeping it below the melting temperature. The proof-of-concept including key characteristics of RLS crystals is demonstrated using the example of Sb_2_S_3_ crystals within the Sb-S-I model glass system for which the rotation rate depends on the direction of laser scanning relative to the orientation of initially formed seed. Lattice rotation in this new mode of crystal growth occurs upon crystallization through a well-organized dislocation/disclination structure introduced at the glass/crystal interface. Implications of RLS growth on biomineralization and spherulitic crystal growth are noted.

All real single crystals deviate from the perfect lattice and contain defects such as vacancies, interstitials, dislocations, etc. In spite of such defects, macroscopic single crystals exhibit long range periodicity and translational symmetry, when characterized by X-ray diffraction or other similar methods. Only when examined on atomic scale, point and line defects are observed by high resolution techniques such as transmission electron microscopy (TEM). In this paper, we consider a different deviation from the perfect lattice in which the lattice orientation gradually rotates. As shown in Fig. 1 this rotating lattice single (RLS) crystal resembles a single crystal that is plastically bent, however, without changing its physical shape. Such gradually rotating lattice has been suggested for several micron size crystals (called “transrotational”), which are formed within amorphous films of Se, Sb_2_S_3_, Sb_2_Se_3_, In_2_Se, Fe_2_O_3_, etc. upon heating directly or with an electron beam^1^,^2^,^3^,^4^. Here the crystal lattice rotates about an axis in the plane of the film, by inasmuch as 120°/μm for crystals those grow as spherulites^5^,^6^,^7^. The resulting crystal dimensions are limited and the growth is uncontrolled. The rotating lattice single (RLS) crystals, if available in large enough size, will form a special class of crystalline solids that can have very unusual and potentially useful electrical, mechanical and optical properties. Note that unlike the twisting and bending of crystal’s macroscopic shape observed in various materials^8^, in the RLS crystal of present interest only lattice rotates while maintaining the macroscopic form.

The gradual change of orientation on a micrometer or larger scale is found in many naturally occurring biominerals^9^,^10^. In this case, however, one encounters polycrystalline material and rotation of nanocrystal orientation. The case of the recently reported biomineral vaterite is especially interesting and relevant^11^, because it exhibits orientation changes between adjacent crystallites within a narrow range of 0 to 30° without any organic interfacial layer that often contributes to the alignment of neighboring crystallites^12^,^13^. This is a completely unexpected result from the classical theory of crystallization by nucleation and growth, and spherulitic growth mode has been suggested to explain these observations. Such spherulitic crystal growth has been observed in a wide range of substances that crystallize from high viscosity melt or solutions^14^,^15^,^16^,^17^,^18^,^19^,^20^,^21^,^22^,^23^,^24^. However, its mechanism, in particular the source of non-crystallographic branching in spherulites remains unclear^7^. So the knowledge of how lattice rotation is introduced is important for understanding the formation of spherulites^1^,^4^,^5^,^6^,^7^,^14^,^15^,^16^,^17^,^18^,^19^,^20^,^21^,^22^,^23^,^24^ as well as biominerals^9^,^10^,^11^,^12^,^13^. The ability to control this rotation will help fabricate ‘bio-inspired’ materials that mimic the structure of biominerals and hence their superior properties^25^.

The observations of rotating lattice in spherulites and our recent success in the fabrication of single crystal architecture (dots, lines and 2D layers) on the surface of a bulk glass^26^ have motivated us to explore the possibility of fabricating large-scale RLS crystals. Indeed the sign of lattice rotation was present but not recognized in the recently reported work;^26^ it became evident only upon a more detailed and precise analysis of structure using micro X-ray diffraction presented here. In this work, an appropriate, focused laser beam provides the fine process control and enables us to “write” narrow crystalline lines of indefinite length within a glass sample. We further demonstrate 2D-RLS crystal structures by scanning several lines next to each other. Since the temperature distribution for fabricating such single crystal architecture is well controlled, the lattice rotation (i.e. rate of rotation per micrometer Θ°/μm) can be varied as desired. Thus we demonstrate structures that are sufficiently large RLS crystals to impact the physical properties through lattice rotation.

Among various materials, we chose Sb_2_S_3_ as a model system because spherulitic microcrystallites have been observed previously during the crystallization of amorphous Sb-S films by electron beam heating^1^,^6^,^27^. To establish the possibility of a RLS crystal over extended dimensions and the degree to which lattice rotation can be controlled we have investigated laser induced crystallization of Sb_2_S_3_ within the Sb-S-I glass forming system.

## Results

To demonstrate the feasibility of extensive RLS crystal two sulfide glass compositions were selected within the Sb-S-I system: congruently crystallizing Sb_2_S_3_ and non-stoichiometric 16SbI_3_–84Sb_2_S_3_. These compositions have been shown to crystallize and form extended single crystal lines and 2D layers when heated locally with a continuous wave (CW) laser via solid state transformation^26^. Fabrication of Sb_2_S_3_ crystal is initiated with the formation of a seed crystal nucleated at or just below the surface of the glass (top of the line in Fig. 2a). The sample is then moved in a straight line at a speed of 1 μm/s while the laser position remains fixed, resulting in a linear 1D crystal as seen in the figure for Sb_2_S_3_ glass. Qualitatively similar results are obtained with the other glass. The remaining panels of Fig. 2 describe the nature of the so formed crystal, which is established from a detailed analysis of the lattice orientation by electron back scattered diffraction (EBSD) mapping.

The complete orientation of the lattice of laser-crystallized regions was determined from EBSD analysis for three mutually orthogonal directions: the so called rolling direction (RD) that coincided with the direction of laser movement on the surface of sample, the normal direction (ND) that was perpendicular to the sample surface, and the transverse direction (TD) that was normal to both RD and ND. The inverse pole figure maps constructed from EBSD patterns for the sample region in Fig. 2a are shown in 2b, 2c and 2d for TD, ND and RD directions, respectively. They indicate the lattice orientation along the directions of the three reference axes. Thus, the RD-map gives the lattice orientation parallel to the line itself, the ND-map gives the orientation in the direction normal to the surface, and the TD-map gives the orientation orthogonal to both. The same color for all regions in Fig. 2b, seed dot as well as the line, suggests that the crystal has the same crystallographic orientation in transverse (TD) direction, viz. <010>. This observation is similar to that reported in reference (26) recently. In contrast to the uniformity of color in 2b, there is a gradual variation in Fig. 2c,d. This gradual change in the orientation of crystal lattice that occurs only along the ND and RD directions and not along TD is further elucidated by the pole figures for <010>, <100> and <001> crystallographic directions in Fig. 2f. A comparison of the various maps indicates that the <001> and <100> orientations of the crystal rotate about the transverse (TD) direction that is close to <010> of the crystal (see pole figure diagrams for these main axes of crystal lattice and space orientations of crystal lattices plotted on IPF ND map). The crystal lattice rotates about an in-plane direction (TD, in our EBSD experimental setting), which is normal to laser scan direction (RD). Figure 2g shows a crystal orientation deviation (COD) map with respect to crystal orientation of seed dot center, amplifying lattice orientation changes. Figure 2h shows the variation of orientation quantitatively as well as initial and final orientation of crystal lattice for the dot and line. The rate of lattice rotation (Θ°/μm) is found to be 0.56 ± 0.02°/μm. The plot (Fig. 2h) shows that once the crystal line is established from the initial seed, the orientation changes linearly with distance. Also from EBSD maps the sign of the rotation depicted in Fig. 2c was obtained. The crystal lattice rotates downward in the line as it is shown on the figure.

The different rates of lattice rotation in the seed region and along the remaining line (Fig. 2h) shows that the dynamic heat profile around the laser spot and the direction of laser scanning relative to initial seed are important parameters that control the rotation of the lattice formed during crystal formation. To further prove this hypothesis we changed the laser scanning direction while the crystal was forming. The results are shown in Fig. 3, where several independent crystal lines were written in different directions starting from the same central position on the first line marked #0 on the surface of 16SbI_3_–84Sb_2_S_3_ glass. The new crystals marked #1, #6 and #7 were formed as straight lines, whereas a bend was added to crystal lines #2, #3 and #5 at a later stage. It is particularly striking that there are some directions for which there may be no rotation of lattice, for example line #5 for which Θ is zero within the experimental uncertainty. For other laser scanning directions, the value is non-zero and depends on the direction of laser scanning. It may change to another value if a bend is created in the direction of crystal growth, as seen for crystal lines #3 and #4. Also the crystal lattice rotates downward in all lines as marked on the lines in Fig. 3b. Figure 3c describes the rotation of various RLS crystal lines quantitatively. The overall straight line variation testifies the gradually rotating lattice of laser-written crystal lines.

To complement the lattice orientation information provided by EBSD that has excellent spatial resolution but with very shallow probed depth (a few nm) and relatively poor angle accuracy (typically, 1–2°), we examined the same sample with scanning X-ray microdiffraction (μSXRD) using polychromatic x-ray beam that has much higher orientational accuracy (typically, ±0.01°), sub-micron spatial resolution and probe depths up to ~100 μm. The evolution of lattice orientation as manifested in Laue spots is shown in Fig. 4. As an illustration, Laue spot corresponding to (852) planes is analyzed in detail. For comparison, Laue diffraction from an unconstrained Sb_2_S_3_ single crystal is shown in the figure. The diffraction from the initial seed of laser-written crystal (point #1 in Fig. 4c) in glass shows four radial extensions of diffuse scattering around the reflection spot (#1 in Fig. 4b). As soon as the laser moves away from the dot (point #2 and #3 in Fig. 4c), the Laue spots become steadily elongated with a rod-like intensity distribution for the whole length of the crystal line (Fig. 4b).

Finally, the versatility of RLS crystal structure is demonstrated by fabricating a 2D single crystal layer on the surface of glass, as seen in Fig. 5a. It is constructed by rastering the laser beam with 10 μm lateral steps on the surface of 16SbI_3_–84Sb_2_S_3_ glass. A previously laser-induced line was used as seed for growth. Figure 5b shows a COD map with respect to crystal orientation of seed. It shows that lattice orientation of the 2D crystal architecture changes gradually (0.05°/μm) from one line to the next line. However, the same colors of individual lines in Fig. 5b suggest that there is no significant change in their orientation during growth. The crystal lattice only rotates downward at every step between the lines, as indicated by color contrast between them.

## Discussion

**Figure 1:**
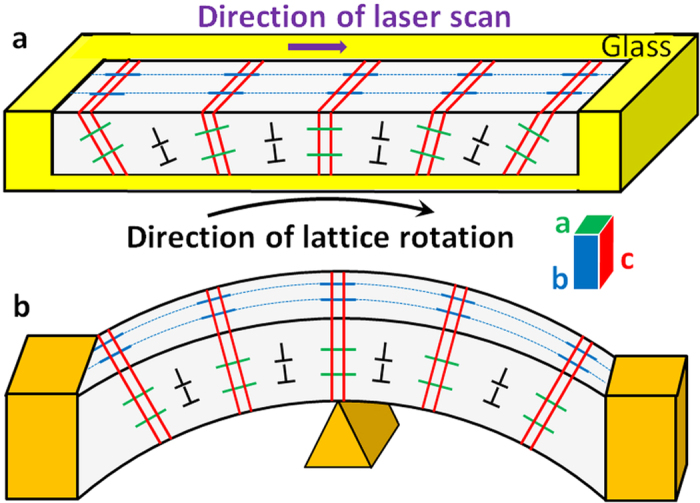
Schematic of different rotating lattice single (RLS) crystals. A crystal fabricated by laser on the surface of a bulk glass (**a**), and another single crystal after plastic deformation (**b**). Green, blue and red lines indicate crossing crystallographic planes <100>, <010> and <001> with top and front edges of crystals in grains divided by dislocation walls. Only six grains are shown for clarity.

**Figure 2:**
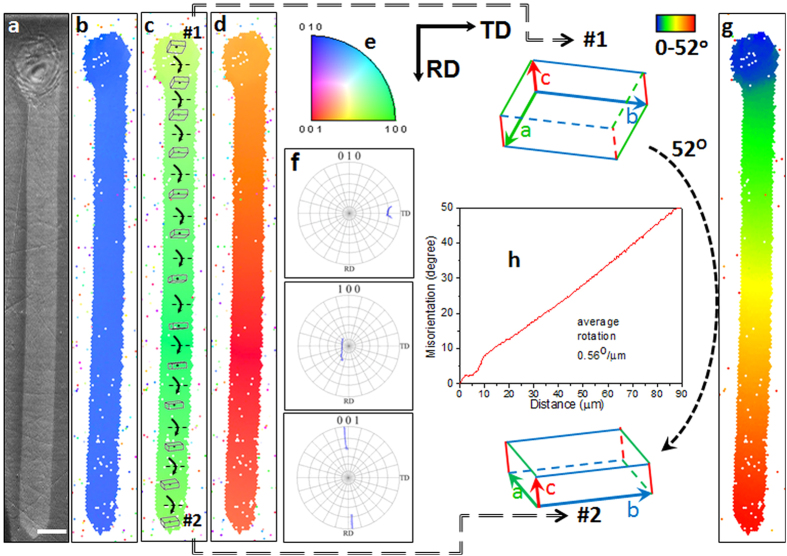
Laser-induced Sb_2_S_3_ RLS crystal line on the surface of Sb_2_S_3_ glass. The laser-induced dot was created by slowly ramping the power density from 0 to 60 μW/μm^2^ in 5 s and maintaining it for 60 s. The subsequent exposure transformed the surface of Sb_2_S_3_ glass to a crystal line by moving the laser spot with scanning speed 1 μm/s. Scale bar corresponds to 5 μm. SEM image (**a**) and colored orientation IPF maps with reference vector in the direction of surface normal TD (**b**), ND (**c**) and RD (**d**) and pole figure for <001>, <100> and <010> directions of Sb_2_S_3_ (**f**) for the crystal (dot and line). Unit cells with varying orientation of the lattice are shown on IPF ND map (**c**), while the crystal orientation deviation (COD) map (**g**) relative to lattice orientation in the dot indicates rotation of Sb_2_S_3_ crystal. The plot (**h**) shows quantitative changes of the lattice orientation with distance from top point of the seed as well as initial and final orientation of the unit cell. The crystal lattice rotates clockwise in the line as the laser beam is moved from left to right. Arrows on IPF ND map (**c**) describe this rotation.

**Figure 3:**
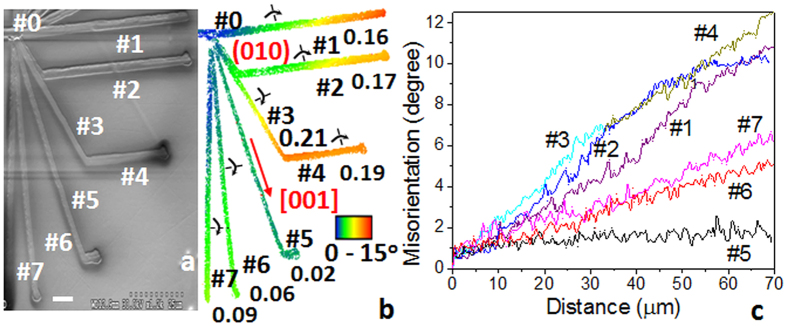
Laser-induced Sb_2_S_3_ RLS crystal architecture on the surface of 16SbI_3_–84Sb_2_S_3_ glass. First, a laser-induced line #0 was created by slowly ramping the power density from 0 to 90 μW/μm^2^ in 5s, followed by steady exposure for 60 s and then by moving the laser spot at a scanning speed of 20 μm/s. Scale bar corresponds to 5 μm. New lines (#1–#7) were formed from the end of the initial line (#0) at 20 μm/s scanning speed, 90 μW/μm^2^ power density and 1s initial exposure. Scale bar corresponds to 5 μm. SEM image is shown in (**a**), and crystal orientation deviation (COD) maps in (**b**) are relative to lattice orientation at the end of line #0. Quantitative changes of the orientation are plotted in (**c**) as a function of distance from the starting point of the lines. Arrows and the numbers on COD map (**b**) describe the axis and direction of the unit cell rotations, and their corresponding rotation rates (Θ°/μm), respectively.

The crystal orientation deviation maps (Figs 2g, 3b and 5b) of the crystals fabricated by laser heating of the glasses in this study clearly show a gradual rotation of crystal lattice. Specifically, the orientation of the unit cell of the Sb_2_S_3_ crystal lattice gradually changes as marked schematically on the crystal line of Fig. 2(c,h). Here, as one moves along the scan direction, the lattice rotates downwards about the axis that is in the plane of crystal, which is also the sample surface, and normal to the direction of laser scanning. We note that the orientation of the crystal line relative to transverse direction, as seen in Fig. 2b, does not change at all. We would consider this line as single crystal if our view was limited only along the transverse direction. A conflict with the formal definition of a single crystal arises only when viewed along the rolling or normal directions (Fig. 2c,d). However, it is noteworthy that even in these directions the rate of rotation Θ °/μm, which is the slope of the straight line in Fig. 2h, is very well defined. As we will see later, these crystal lines can be described by introducing dislocation/disclination defects in an otherwise perfect single crystal. Since real crystals with such defects are usually considered single crystals, we will continue to call the structures of Fig. 1a as single crystals, but with a rotating lattice given by Θ.

We have demonstrated precisely the fabrication of RLS crystals of unlimited length, which we had hoped to obtain in this work. Unlike previous observations made within small grains, the present RLS crystal lines can be extended to effectively any length. More importantly, the rate of the rotation Θ in the present case is constant and smooth along the length of the crystal. Even though the value of Θ is more than an order of magnitude smaller than in prior observations on small grains^2^,^4^, it spans a large range from effectively zero to 0.6°/μm. These characteristics make such crystals interesting for the fundamental understanding of the transformation of glass to crystal as well as useful in practical applications.

**Figure 4:**
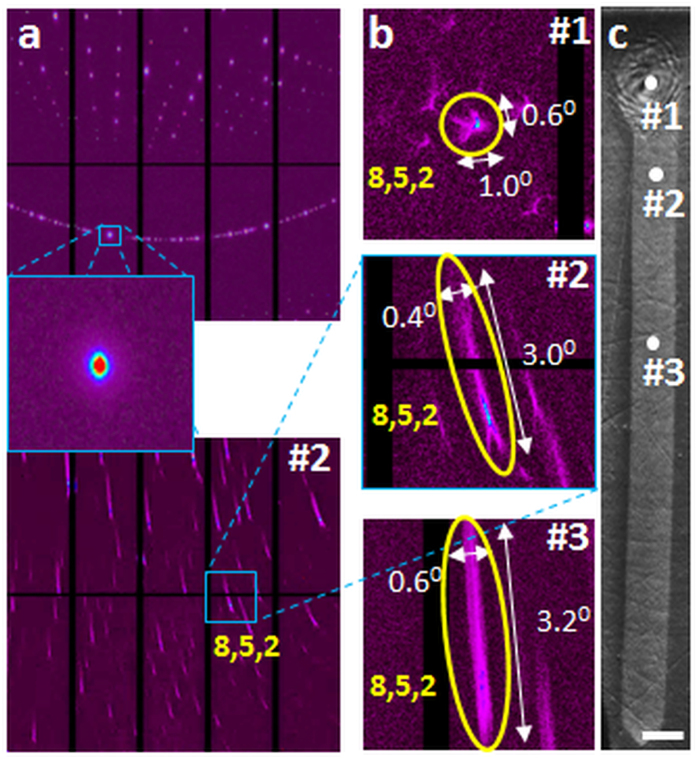
Results of scanning X-ray microdiffraction (μSXRD) with submicron spatial resolution. Laue diffraction (**a**) from an unconstrained Sb_2_S_3_ single crystal (top) and laser fabricated RLS crystal Sb_2_S_3_ (bottom). Magnified images (**b**) of selected reflection (852) extracted from Laue patterns (a, bottom) obtained for different points of the RLS crystal (**c**), shown in Fig. 2.

A detailed insight about the mechanism of lattice rotation within RLS crystal lines is obtained from Laue microdiffraction results shown in Fig. 4. In comparison to the Laue pattern for free-standing single crystal, which comprises of well-formed circular spots (Fig. 4a), the corresponding Laue spot of the laser-formed RLS crystal are more complex. The Laue spots from the regions within a crystal line exhibit extensive streaking (see Fig. 4b). The origin of these streaks can be elucidated by comparison with other systems such as nanoindented copper single crystals^28^, and deformed individual crystalline grains of nickel^29^. In these cases, the material deforms plastically under stress through the nucleation, movement and interaction of dislocations to accommodate the strain^30^. These specific rearrangements of the dislocations include the pile-ups of same sign dislocations into small angle (***ω***) boundaries. According to the definition of Volterra^31^, such dislocation assemblies (tilt dislocation walls, TDW) can be considered also as wedge partial disclinations defined by corresponding Frank rotation vector ***ω***^32^. These wedge disclinations have been observed directly by TEM and EBSD of plastically deformed metallic materials^33^,^34^ as well as in spherulitic Sb_2_S_3_ crystals in amorphous films crystallized by electron beam heating^1^.

The Laue patterns in Fig. 4 for the single crystal line in Fig. 2 are very similar to those simulated by Barabash *et al.* for crystals that comprise of both tilt dislocation walls and individual geometrically necessary dislocations (GNDs)^28^,^35^. Using this description we attempt to explain the macroscopic lattice rotation in the line as indicated by the EBSD maps in Figs 2 and 3. Our structural model of RLS crystal is shown schematically in Fig. 6. Effectively, our RLS crystal (Fig. 1a) is similar in structure to a “plastically deformed single crystal” (Fig. 1b), wherein a disclination produces small abrupt rotation (***ω*** < 1^0^) and unpaired GNDs introduce a more gradual rotation of crystal lattice. However, there is a fundamental difference between the plastically deformed and laser-written RLS crystals. Under applied forces the initially perfect single crystal changes its shape, for example, from rod to bent arc (see Fig. 1b). On the other hand, the straight RLS crystal lines are not bent macroscopically, at all. The reason for the difference between the two crystals is in their respective origins of lattice rotation: in the plastically deformed single crystal, the lattice is rotated in response to the applied stresses. By contrast, lattice rotation in a RLS crystal line occurs upon solid-state crystallization of glass and it takes place without a change of the shape.

A situation rather closely related to the growth of RLS crystal is the helical growth of hippuric acid in its undercooled melt^14^. Its crystals grow as needles in which lattice twists about the axis of the needle. They grow isothermally in contrast to RLS crystals that grow under very high temperature gradients, and their lattice can untwist under the influence of elastic stresses during growth. The lattice twisting is believed to result from the introduction of screw dislocations, which parallels lattice rotation in RLS crystals but via edge dislocations.

For a more quantitative explanation of the lattice rotation in our RLS crystal, we need to take into account that the underlying process occurs effectively in the solid state and does not occur during growth from the melt, as noted by Stone *et al.*^36^ for laser-induced transformation of LaBGeO_5_ glass deep inside the sample to a single crystal of the same composition. We note that under the conditions of our experiments, the RLS crystal growth is a dynamic solid → solid transformation that is constrained simultaneously by the structures of the crystal preceding the growth front on one side and of the surrounding glass on the other side of the front. The transformation requires rearrangement of atoms, which must diffuse short distances if there is no change in composition, or long distances in the case of incongruent crystallization. Consequently, we can expect that the structure of both the glass and the crystal, at least in the vicinity of interface, changes and influences the structure of the other side. The relative magnitude of the changes on the two sides will depend on local viscosity, shear modulus and generally the resistance to deformation. The relatively more open structure and supercooled state should allow greater percentage changes on the glass side than on the crystal side, although the amorphousness of its structure may make the observation of such changes difficult. In any case, the glass structure should change concurrently for the same reason the crystal lattice undergoes systematic rotation.

To understand the growth of a crystal at a crystal/glass interface, we may draw an analogy to hetero-epitaxial crystal growth such as of GaN on sapphire^37^, when misfit dislocations appear periodically to compensate for the lattice parameter mismatch. Additional half-plane appears on the side of crystal with smaller lattice parameter (see Fig. 6, insert A). In the case of glass-crystal interface obviously we expect mismatch due the difference in the structure and density of the two regions. At this interface misfit dislocations of one sign are generated preferentially to compensate the larger molar volume of amorphous phase (see Fig. 7). Consequently, such unpaired dislocations, initially appearing at the interface, continue to propagate in the volume of growing crystal. Dislocations of opposite sign are not energetically favorable.

Following the formation of dot seed, as the laser beam traverses from left to right, crystal-glass front follows the beam. As an example, using the data of Fig. 6 for Sb_2_S_3_ crystal line with Θ = 0.56°/μm (as determined from EBSD) and the elongation of its Laue reflection spot by 3.2°, we estimate the thickness of the crystal into the substrate approximately 1.5 μm. We consider two qualitatively different crystallization scenarios depending on whether the peak of the growth front is at: (i) the sample surface (Fig. 7a), or (ii) below the surface (Fig. 7b). In the first case, the crystal grows under asymmetric confinement by the surrounding structure, which introduces dislocations in the crystal side at the interface (Fig. 7a). Specifically, unpaired edge dislocations are added with extra half planes from the top side (free crystal surface), which induce clockwise lattice rotation. The rate of lattice rotation i.e. magnitude of Θ is determined by the ease with which such dislocations are introduced relative to the crystal growth rate (that itself depends on the direction of laser motion relative to lattice orientation). The so-formed crystal lattice would appear rotated to an external observer. In the latter case, the crystal-glass interface remains symmetrical relative to scanning direction and dislocations of opposite signs are introduced at the opposite sides, so that there is no net rotation of the lattice. Such a single crystal is realized by laser-induced transformation of glass deep inside the sample^36^.

Note that unpaired dislocations can be introduced spontaneously at the crystal-glass interface, as in the case of hetero-epitaxial growth. Alternatively, the glass itself can help nucleate the dislocation/disclination structure into the crystal, if we consider the disclination model of glass-crystal interface proposed by Ovidko *et al.*^38^,^39^. Here a glass is viewed as a random distribution of disclinations, which destroy the long range order while retaining low-energy configurations related to the crystal structure^40^,^41^. Then the amorphous phase contains straight lines of wedge disclinations normal to crystal-glass interface. The wedge disclinations of small strength (i.e. small rotation angle ***ω***) and line vector normal to scanning (crystal growing) direction of laser beam (see Figs 6 and 7) would extend from glassy phase into the crystal across the interface^38^,^39^.

The fundamental reason for the presence of above described dislocation/disclination structure and the cause of observed rotation of the lattice of RLS crystal is the difference in the structure of the crystalline phase and precursor glass. As the glass transforms into a crystal under laser heating, there is a concomitant decrease in volume, which would in principle place the crystal under tension and the surrounding glass under compression. Under the special solid state single crystal growth process realized only recently^26^ and employed in this study, the crystal is well below the melting temperature. Then the easiest way for it to absorb the tension is via formation of misfit dislocations. Since the mismatch between the glass and crystal structures across their interface occurs during the crystal growth process, the dislocations would be introduced then and there. Any stresses introduced from thermal contraction mismatch during cooling from the crystal growth temperature to the ambient are expected to be small for two reasons: (i) there has been no observable change in shape, and (ii) the direction of lattice rotation due to such stresses will be opposite to what is observed here (note: thermal expansion coefficient of glass is usually larger than that of crystal of same composition).

**Figure 5:**
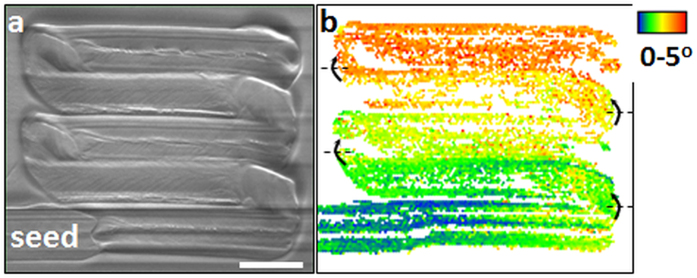
The 2D single crystal pattern created by rastering the laser beam along the length of line followed by a 10 μm transverse step on the surface of 16SbI_3_–84Sb_2_S_3_ glass. SEM image (**a**) and colored crystal orientation deviation (COD) map (**b**). The previously laser-induced line was used as seed for growth. Scale bar 10 μm. Arrows on COD map (**b**) describe the axis and direction of the lattice cell rotations during transverse step.

**Figure 6:**
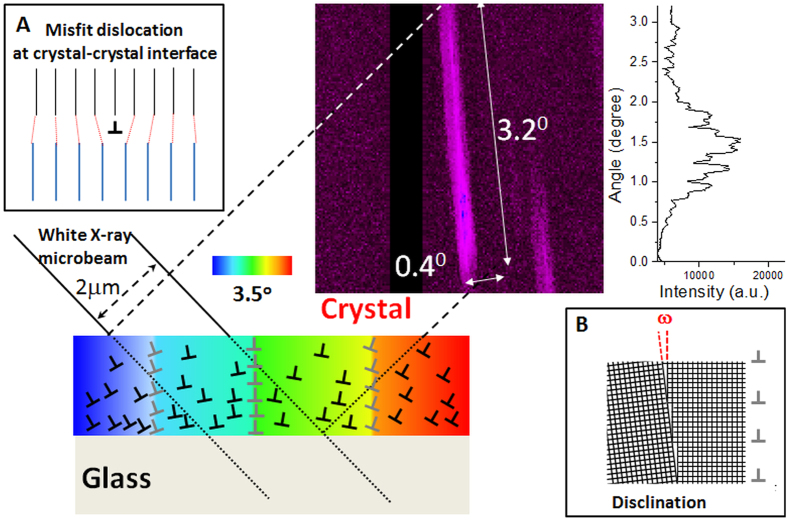
Explanation of elongated Laue reflection observed for Sb_2_S_3_ crystal grown on the surface of glass matrix. The crystal contains unpaired edge dislocations (such as misfit edge dislocation on crystal-crystal interface as seen in insert (**A**), and tilt dislocation walls or wedge disclinations (such as seen in insert (**B**). Disclinations produce small abrupt rotation (ω~1^0^), whereas unpaired dislocations introduce gradual rotation of crystal lattice. The overall crystal rotation is shown by color gradient.

**Figure 7:**
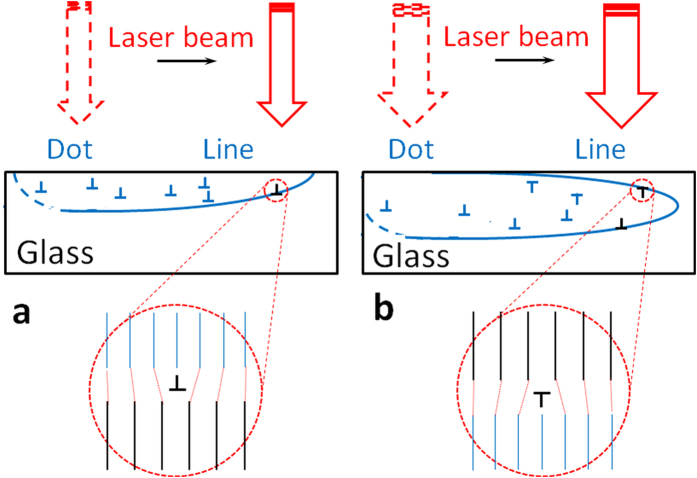
Schematic of the dislocation model of RLS crystal growth. A crystal fabricated by laser heating when the leading edge of the growth front is at the sample surface (**a**), or below the surface (**b**).

An intriguing observation in the present study is that of effectively no lattice rotation for crystal line #5 in Fig. 3 or along the lines of the 2D crystal in Fig. 5. Crystal structure of Sb_2_S_3_ has orthorhombic symmetry with unit cell dimensions a = 11.25 ± 0.02 Å, b = 11.33 ± 0.02 Å, c = 3.84 ± 0.01 Å^42^. Such an Sb_2_S_3_ crystal line was written on the surface of 16SbI_3_–84Sb_2_S_3_ glass in the crystal’s (010) plane along <001>, which is also the direction of fastest crystal growth^43^. This orientation of crystal is consistent with the (010)/[001] primary slip system experimentally established for natural Sb_2_S_3_ crystals^44^, which is, as expected, also the direction of shortest bonds (i.e. most dense packing) on the closest packed planes for its crystal structure^42^,^45^. So one would expect maximum mismatch at this particular line’s advancing interface with the glass matrix. Apparently, for this specific orientation the crystal is most resistant to accept or generate dislocations, so that all the mismatch of transformation is accommodated effectively in the surrounding amorphous structure. Then the crystal grows without significant number of unpaired dislocations or disclinations, resulting in zero lattice rotation.

It is instructive to compare the RLS crystal growth with spherulitic crystal growth that is observed in a broad range of materials and under very different conditions. Here, the crystal grows radially outward, and lattice rotation along with defects accommodates up to several percent increase in density upon crystallization. Branching of crystals in spherulites is very common, which represents significant differences in the orientations of adjacent crystals. It may appear similar to the presence of TDWs that separate neighboring subgrains in RLS crystal lines. Indeed, high resolution transmission electron microscopy has revealed that the micro-crystallites within spherulitic crystals comprise of dislocations and small angle boundaries^1^,^5^. However, there is also a key difference between the two processes: in a spherulite, the lattice is rotated in response to the requirement of crystal growth in all directions in 2D (even in 3D) starting at a point. Indeed, such form of crystal growth occurs during the formation of initial dot that serves as the seed for fabricating crystal line in Fig. 2. The Laue spots in the μSXRD pattern of this region consists of diffuse scattering with fourfold symmetry (#1 in Fig. 4b), which indicates the presence of four different sets of oriented dislocations within the scattering volume of the dot. By contrast, lattice rotation in a RLS crystal line occurs in a narrowly defined direction – practically in 1D. The present observations suggest that the volume change associated with amorphous → crystal transformation and the influence of disclination structure of amorphous phase on the defect structure of crystal should also be considered explicitly in establishing a comprehensive mechanism of spherulitic crystal growth.

Finally, we note examples of rotating orientation of crystallites in biominerals such as vaterite in nature^46^. A common precursor of such crystal growth, as in the present RLS crystals, is the presence of a surrounding amorphous phase. We propose that the growth of such biominerals also occurs via a process analogous to RLS crystal growth, where amorphous CaCO_3_ forms first, followed by crystallization, rather than direct crystallization from the solution phase^47^,^48^,^49^. Indeed Pokroy *et al.*^11^ hypothesized such a crystal growth mechanism, and the present work provides experimental support and extends the basic idea to biomineralization in the absence of intervening organic layer.

In conclusion, the present investigation has clearly demonstrated the concept and realization of RLS crystals of practically unlimited size by exploiting laser-induced solid state transformation of glass to single crystal. The absorption of the focused laser light at or near the surface provides conditions for asymmetric crystal growth, which introduce unpaired dislocations and wedge disclinations at the glass-crystal interface. The rate of lattice rotation, which for the investigated Sb_2_S_3_ crystals varies from zero to 0.6 °/μm, is determined by the direction of laser movement relative to lattice orientation. The laser-heating process developed here is well controlled, allowing the fabrication of RLS crystal with a variety of lattice rotation rates and arbitrary shapes, including straight or bent lines, 2D patterns, etc. Since the rotation of lattice is accomplished by incorporating dislocations and disclinations in the structure, the present fabrication method provides a method for engineering such defects without requiring plastic deformation or shape modification.

## Materials and Methods

### Glass Preparation

The two sulfide glasses of this study, congruently crystallizing Sb_2_S_3_ and non-stoichiometric 16SbI_3_–84Sb_2_S_3_, were made following the ampule quenching method previously developed for the Sb-S-I system^50^. To make Sb_2_S_3_ samples, which does not form glass easily, the melt cooling rate was increased by limiting quartz ampules to 1 mm ID and 10 μm wall thickness. The 16SbI_3_–84Sb_2_S_3_ glass was prepared using ampules with 11 mm ID and wall thickness 1 mm. X-ray diffraction analysis of the as-quenched samples confirmed their amorphous state. The samples for laser-induced treatments were polished using metallographic techniques.

### Laser-induced crystallization

A fiber-coupled 639 nm diode laser (LP639-SF70, ThorLabs) was used for crystallization. Its intensity was modulated by an analog voltage (ILX Lightwave LDX-3545 Precision Current Source). The beam was focused on the polished surface of the glass sample by a 50x, 0.75NA microscope objective to a spot of a few μm diameter, and the crystalline lines a few hundred μm long were fabricated by translating the sample at a fixed power density. The sample was placed in a flowing nitrogen environment on a custom-built stage, which could be translated independently in the x-, y-, and z-directions. A CCD camera monitored the sample *in-situ*, while LabView software controlled the laser intensity, and the movement of the stage. The optical setup and procedures for preparation of the glass samples and fabrication of crystals have been described in detail in previous publications^26^,^51^,^52^,^53^.

### Materials characterization

The laser-irradiated regions were analyzed by a scanning electron microscope (SEM, Hitachi 4300 SE) in water vapor environment to eliminate charging effects. The chemical compositions were determined at multiple locations on each sample by EDS detector attached to SEM, using the EDAX-Genesis software. Local crystallinity and orientation were determined by EBSD with Kikuchi patterns collected by a Hikari detector within the SEM column. EBSD pattern scans were collected and indexed using TSL OIM Data Collection software, whereas Orientation Imaging Microscopy Analysis software yielded image quality, pole figure and inverse pole figure maps^54^. The crystallinity and orientation of the laser created dots and lines were further examined by scanning Laue X-ray microdiffraction (μSXRD) with submicrometer spatial resolution on beamline 12.3.2 of the Advance Light Source synchrotron, Lawrence Berkeley National Laboratory^55^. Polychromatic x-ray beam with an energy range of 6–22 keV was focused to submicron size via a pair of elliptically bent x-ray mirrors in a Kirkpatrick-Baez configurations. Samples were raster scanned under the x-ray beam with a step size of 1 micron. At each step a Laue pattern was collected using a DECTRIS Pilatus 1 M hybrid pixel detector. Indexing of the Laue patterns was completed using the XMAS software^56^.

## Additional Information

**How to cite this article**: Savytskii, D. *et al.* Rotating lattice single crystal architecture on the surface of glass. *Sci. Rep.* **6**, 36449; doi: 10.1038/srep36449 (2016).

**Publisher’s note:** Springer Nature remains neutral with regard to jurisdictional claims in published maps and institutional affiliations.
